# Evaluation of the Time Spent by Anesthetist on Clinical Tasks in the Operating Room

**DOI:** 10.3389/fmed.2021.768919

**Published:** 2022-01-17

**Authors:** Vincent Compère, Emmanuel Besnier, Thomas Clavier, Nicolas Byhet, Florent Lefranc, Frederic Jegou, Nicolas Sturzenegger, Jean Baptiste Hardy, Bertrand Dureuil, Thomas Elie

**Affiliations:** ^1^Department of Anaesthesiology and Intensive Care, Rouen University Hospital, Rouen, France; ^2^Normandie Université, UNIROUEN, INSERM U982, Mont-Saint-Aignan, France; ^3^Department of Anaesthesiology, Dieppe General Hospital, Dieppe, France; ^4^Department of Anaesthesiology, Hôpital privé de l'estuaire, Le Havre, France; ^5^Department of Anaesthesiology, Clinique du Cèdre, Bois-Guillaume, France; ^6^Department of Anaesthesiology, Evreux General Hospital, Evreux, France

**Keywords:** clinical tasks, efficiency, anesthesia organization time, interruption task, anesthesia

## Abstract

**Background:**

Changes in the health system in Western countries have increased the scope of the daily tasks assigned to physicians', anesthetists included. As already shown in other specialties, increased non-clinical burden reduces the clinical time spent with patients.

**Methods:**

This was a multicenter, prospective, observational study conducted in 6 public and private hospitals in France. The primary endpoint was the evaluation by an external observer of the time spent per day (in minutes) by anesthetists on clinical tasks in the operating room. Secondary endpoints were the time spent per day (in minutes) on non-clinical organizational tasks and the number of task interruptions per hour of work.

**Results:**

Between October 2017 and April 2018, 54 anesthetists from six hospitals (1 public university hospital, two public general hospitals and three private hospitals) were included. They were followed for 96 days corresponding to 550 hours of work. The proportion of overall clinical time was 62% (58% 95%CI [53; 63] for direct care. The proportion of organizational time was higher in public hospitals (11% in the university hospital (*p* < 0.001) and 4% in general hospitals (*p* < 0.01)) compared to private hospitals (1%). The number of task interruptions (1.5/h ± 1.4 in all hospitals) was 4 times higher in the university hospital (2.2/h ± 1.6) compared to private hospitals (0.5/h ± 0.3) (*p* < 0.05).

**Conclusions:**

Most time in the operating room was spent on clinical care with a significant contrast between public and private hospitals for organizational time.

## Highlights

Most time in the operating room was spent on clinical care 62 % (58 % direct care and 4 % indirect care) with a significant contrast between public and private hospitals for organizational time (11% in the university hospital and 4% in general hospitals compared to 1% in private hospitals).

## Introduction

Changes in the health system in Western countries have increased the scope of the tasks assigned to physicians in their daily lives. The burden of administrative tasks reduces the time spent with the patient. A recent work showed that in four different specialties (general medicine, internal medicine, cardiology and orthopedics), for every hour spent with a patient, a physician spent 2 h on tasks in the patient's absence (1). This result is consistent with that of the study of Wenger *et al*. which found a ratio of 1 to 3 for time spent with the patient vs. administrative tasks in a population of 36 internal medicine residents (2).

Anesthetists are confronted with the same reality as other specialties but no recent work has investigated this topic. A first study published in 1976 showed that anesthetists spent most of their time in contact with their patients: in direct observation (e.g. auscultation) or indirect observation (monitoring of constants) and adapting anesthesia drugs (3). A decade later, McDonald *et al*. reported a clinical time of 61% with 17% directly with the patient (4). In German multicentric study published in 2011, anesthesiologists spent 28.5% of each workday on indirect patient care, 14.7% on direct patient care and 18.8% on administrative work (5). Communication took up 19.9% of anesthesiologists' time, breaks and disruptions 15.2% and other job tasks 2.9%. The time spent on other non-clinical tasks (administrative, organization, etc) appears to be similar than specialties other than anesthesia (20% in the work of Sinsky *et al*.) (1).

A correlation has been shown between the importance of computer tasks in everyday routine and the occurrence of burnout (6). A reduction in clinical time with the patient could be a major source of dissatisfaction for physicians, which can lead to burnout. Anesthetists are more and more confronted with organizational tasks in the operating room that reduce their time spent with patients. No recent work has specifically evaluated the proportion of time spent by anesthetists on clinical and non-clinical tasks in the operating room. The primary objective of this social study was to evaluate the time spent per day by anesthetists on clinical tasks in the operating room. Secondary objectives were the time spent per day on non-clinical organizational tasks and the number of task interruptions per hour of work.

## Materials and Methods

### Study Model

We conducted a multicenter, prospective and observational study in six hospitals in Normandy, France: one university hospital (Rouen University Hospital), two general hospitals (Dieppe General Hospital and Evreux General Hospital) and three private hospitals (Clinique du Cèdre, private hospital in Bois-Guillaume, Hôpital privé de l'estuaire, private hospital in Le Havre and Clinique Pasteur private hospital in Evreux). Fifty-four anesthetists were eligible and volunteered to participate in the study.

### Study Protocol

We evaluated the time spent by anesthetists on clinical tasks during 1 day in the operating room. Each day between 8 a.m. and 3 p.m., an anesthetist, who had been selected the day before, was continuously monitored by an external observer, a student nurse anesthetist who was carrying out a research internship. There were 13 external observers, each of whom was followed up over the 3 weeks of the internship. All external observers had a 1-h training session by an anesthetist (TE) to explain the purpose of the study and the use of software for monitoring tasks. The training session consisted in a dedicated program on how to define and categorize clinical tasks and how to use recording devices. At the end of the training session, they were evaluated to assess their good comprehension of the different information. The information was also available on electronic devices. In case of difficulty, the two investigating anesthetists (TE and VC) were available at any time.

### Organization of the Operating Rooms of Participating Hospitals

The three public hospitals (Rouen University Hospital, Dieppe General Hospital and Evreux General Hospital), operated with one nurse anesthetist in each operating room. An anesthetist was always in charge of two operating rooms. The organization was different according to the type of public hospital. The university hospital has specialty operating rooms (cardiac surgery, digestive and urological surgery, neurosurgery, pediatric surgery, orthopedic surgery and vascular and thoracic surgery) as well as shared operating rooms for emergency and ambulatory surgery, while the two general hospitals each have shared operating rooms integrating all specialties. The three private hospitals each have shared operating rooms. In the three private hospitals, an anesthetist was in charge of two operating rooms but there was only one nurse anesthetist for two operating rooms.

### Data Collection

The data were anonymously collected using a digital tablet with atracker? software. This software was previously configured to monitor 15 items divided into five categories (Table 1): clinical time; non-clinical time; time spent on communication media to perform tasks; time spent in different locations; the number of task interruptions.

**Table 1:** Description of tasks.

**Categories and daily tasks**	**Description**
**Clinical time**	
**Direct care** (All tasks directly related to patient care)	- Direct communication with patient - Monitoring machines - Performing medical procedures
**Indirect care** (all tasks indirectly related to patient care)	- Searching for a patient's medical record - Preparing for a procedure
**Pedagogical time**	Time spent training residents
**Non-clinical time**	
Personal time	Breaks and lunch
Organizational time	Flow management in the operating room, staff meeting for patient programming
Administrative time	Meetings, scheduling, etc.
Other time	Any time not previously defined
**Communication media**	
Paper	Time spent writing on paper
Telephone	Time spent on the professional telephone
Computer	Time spent on the computer for professional tasks
**Location**	
Medical office	Time spent in the office
PACU	Time spent in the PACU
Operating room	Time spent in the operating room
Other	Time spent in the staff room, rest room etc.
**Task interruption**	
	Number of task interruptions

*PACU, post anesthesia care unit*.

When the anesthetist started one of the tasks listed in the software, the external observer clicked on the corresponding task, triggering a stopwatch that stopped as soon as the observer clicked on it again. Several tasks could be followed in parallel. At the end of the day, data were automatically classified by the software and then sent in the form of a table on an Excel database.

For each anesthetist, observers recorded: age, the number of patients cared for during 1 day, the hospital, a satisfaction score of 0 to 10 filled by the anesthetist.

### Study Outcomes

The primary endpoint was the evaluation by an external observer of the time spent per day (in minutes) by anesthetist on clinical tasks in the operating room.

Secondary endpoints were: the time spent per day (in minutes) on non-clinical organizational tasks; the proportion of time spent in different locations; the proportion of time spent on different communication media; the number of task interruptions per hour of work and stakeholders responsible for task interruptions; and anaesthetists' satisfaction of their working day. The definition used for task interruption was the unexpected cessation of human activity, temporary or permanent. The reason could be specific to the operator or, on the contrary, be external to him.

### Ethical Considerations

The protocol was validated by the ethics committee for non-interventional research of Rouen University Hospital (E2017-27) and was registered in clinical trials (NCT03446482). The requirement for written informed consent was waived by the Committee.

### Statistical Analysis

As described by Hauschild *et al*., we wanted to include at least 500 h of anesthesiologist work in a minimum of five different hospitals (5). The values are presented as a mean (± standard deviation) for the characteristics of the study population. The results are presented in proportion, rounded to the nearest unit for the main results. The percentages (IC95%) expressed correspond to the time spent for each item per day and per anesthetist reported on the total observation time per physician. The number of task interruption is expressed as an average of the number of events per hour (± standard deviation). The different parameters were compared using the Kruskall Wallis test and Bonferroni correction. The correlation coefficients were calculated from the Pearson correlation test. We considered an alpha risk of 5%. The data were analyzed using Microsoft Excel ^?^, XLSTAT^?^ and Prism ^?^ software and the biostaTGV website.

## Results

### Characteristics of Population

Between October 2017 and April 2018, 54 anesthetists from six hospitals were included. They were followed for 96 days corresponding to 550 h of assessed work hours. The characteristics of anesthetists according to hospitals are summarized in Table 2. The sex ratio was 2.6.

**Table 2:** Characteristics of anesthetists population.

	**Anesthetists *n***	**Age years**	**Treated patients *n*/day**
**Overall**	54	39 (±10)	8 (±4.6)
**General hospitals**	13	44 (±11)^#^	8 (±3)
**Private hospitals**	14	43 (±8)	10 (±5.6)
**University hospital**	27	35 (±8.6)^*^	6.7 (±4.3)^*^

*Results are presented as means (± standard deviations) or absolute values*.

* *p < 0.05 corresponding to a significant difference between the university Hospital and private hospitals*.

# *p < 0.05 corresponding to a significant difference between the university hospital and general hospitals*.

### Evaluation of the Proportion of Time Spent on the Different Tasks

The proportion of time spent on clinical tasks was 58% 95%CI [53; 63]. The results are summarized in Table 3. The different stakeholders responsible for task interruptions are displayed in Table 4.

**Table 3:** Time spent by anesthetists on clinical and non-clinical tasks in the operating room.

	**Total**	**University hospital**	**General hospitals**	**Private hospitals**
Anesthetists (n)	54	27	13	14
**Task % [95%CI]**				
Direct care	58% [53; 63]	53%^***^ [47; 59]	48% [40; 56]	78%^+++^ [71; 85]
Indirect care	4% [2.7; 5.4]	4% [2.2; 5.8]	2% [0.4; 3.6]	7%^+^ [3.3; 10.7]
Pedagogical time	4% [2.5; 6.2]	7%^**^ [4.7; 9.3]	4% (2, 10)	<1%
Administrative time	4% [1.2; 5.9]	5% [0.5; 9.5]	1% (1, 3)	2% [0.3; 3.7]
Organizational time	7% [4.8; 9]	11%^***^ [7.6; 14.4]	4%^##^ [0.3; 7.7]	1% [0; 2]
Personal time	14% [11.9; 5.3]	14% [11.3; 16.7]	15% [12.3; 17.7]	11% [7.5; 14.5]
Other time	9%	6%	26%	0
**Communication** **media % [95%CI]**				
Computer	7% [5.2; 9.7]	5%^***^ [3.2; 6.8]	1%^##^ [0.2; 2.8]	17%^+++^ [12; 22]
Telephone	2% [1.7; 2.9]	3%^*^ [1.4; 4.6]	1%^##^ [0.4; 1.6]	2% [1.1; 2.9]
Paper	4% [2.7; 4.8]	5%^***^ [3.7; 6.3]	5% (2, 8)	<1%^+^
**Location %** **[95%CI]**				
Operating room	74% [69; 79]	77% [69; 85]	58%^##^ [49; 57]	83%^+++^ [73; 93]
Medical office	6% [3.2; 8]	4% [1.2; 6.8]	13%^##^ (6, 20)	2%^++^ [−0.1; 4.1]
PACU	13% [8.7; 15.6]	12% [4.4; 19.6]	16% [9; 23]	10% [2.7; 15.3]
Other	7% [3.7; 10.2]	7% [1.7; 12.3]	13% [5; 21]	5%^+^ [−0.1; 6.1]
**Task interruption** n/hour	1.5 (±1.4)	2.2 (±1.6)^*^	0.6 (±0.6)^#^	0.5(±0.3)
**Satisfaction**	7.2 (±1.6)	6.7(±1.7)^**^	7.4 (±1.8)	8 (±0.8)

*Results are presented as percentages corresponding to the averages of proportions of anaesthetists' follow-up time for tasks, location and support and as an absolute value corresponding to the averages over the total observation time for interruptions and satisfaction*.

* *Corresponding to a difference between the university hospital and private hospitals*.

# *Corresponding to a difference between the university hospital and general hospitals*.

+ *Corresponding to a difference between the general hospitals and private hospitals*.

*, #, + *p < 0.05*,

**, ##, ++ *p < 0.01*,

***, ###, +++ *p < 0.001*.

**Table 4:** Stakeholders responsible for task interruptions.

**Anesthetist**	**Nurse**	**Nurse anesthetist**	**Medical doctor**	**Resident**	**Surgeon**	**Surgical nurse**	**Unknown**
3%	22%	15%	14%	4%	3%	7%	32%

### Correlation

There was no correlation between anaesthetists' job satisfaction and the number of patients cared for (r = 0.19, 95%CI[−0.03; 0.38], *p* = 0.08). Similarly, the correlation between the satisfaction score and the time spent in the patient's presence was not significant (r = 0.18, 95% CI[−0.03; 0.39], *p* = 0.09).

The correlation between the satisfaction score and the number of task interruptions during the day was significant and inversely proportional (r = −0.28, 95%CI[−0.46; −0.07], *p* = 0.009). The correlation between the satisfaction score and organizational time was significant and inversely proportional (r = −0.34, 95%CI[−0.52; −0.13], *p* = 0.002). The correlation between the satisfaction score and computer time was significant and positive (r = 0.26, 95%CI [0.05; 0.45], *p* = 0.02).

## Discussion

In this work based on an evaluation by external observers of the tasks in the operating room of 54 anesthetists corresponding to 550 h of follow-up in six centers, the overall clinical time of anesthetist was 62 % (58 % direct care and 4 % indirect care). This result is the same as that of an older work, published by Kennedy et al. in 1976, which focused on the proportion of time spent on the different tasks of the anesthetists during one day in the operating room. anesthetists spent most of their time in contact with their patients in the operating room to monitor and adapt anesthesia (>50%). These authors reported that anesthetists spent too much time (>30%) on tasks considered secondary (data recording, equipment preparation, etc.) because not related to direct care but which could be included in our definition of clinical time (3). About 10 years later, Mc Donald *et al*. evaluated the division of tasks of anesthetists in the operating room in order to analyze the impact of the modernization of monitoring on time spent in direct care. These authors found that 17% of anaesthetists' time was spent directly observing patients and about 40% on ancillary tasks (data recording, preparation, etc.) or observing the monitoring screens. However, if we add the clinical tasks related to anesthesia, the overall proportion of time spent on care was more than 70%, higher than our results (4). It is difficult to compare the results of our work with these two studies because of major changes in the practice of anesthesia over the past 30 years. Similarly, the definition of the tasks they used were not fully comparable with ours. The most recent wok shows that German anesthesiologists spent 28.5% of each workday on indirect patient care, 14.7% on direct patient care and 18.8% on administrative work (5). The type of hospital could change the ratio between different tasks. Indeed, Dexter *et al*. found 53.2% for direct clinical care, 11.5% for indirect clinical care, 10.3% for education and 12.9% for management (7).

Nevertheless, these results relative to ours suggest a continuous decrease in clinical time over the years. Our results are also consistent with those of different studies that focused on other specialties. In the study by Sinsky et al., the clinical time in the presence of patients was 33% in ambulatory practice (family medicine, internal medicine etc.) (1). Wenger et al. found similar results in a population of medical residents The authors attributed the decrease in clinical time to the increasing importance of computer tools (2). In an intensive care unit, the proportion of direct care ranged from 16 to 19% (8). Our results show that, on average, anesthetists spend 2–3 times longer on direct care than other medical specialties. One of the reasons is that in the French model, anesthetists are in charge of two operating rooms and are therefore responsible for two patients at the same time.

In our work, we found a significant difference in the time spent on direct care between public (48 and 53% for general hospitals and the university hospital, respectively) and private hospitals (78%). There are several explanations for this result. First, in the private hospitals observed, the anesthetist worked with only one nurse anesthetist for two rooms, which required the anesthetist to be present continuously in one of the two operating rooms and therefore the time in the presence of the patient was higher. In addition, in public hospitals, particularly in university hospitals, time is spent training residents (7%), which is not the case in private hospitals. Finally, this work highlights the higher organizational constraints in public hospitals (11% in the university hospital and 4% in general hospitals) compared to private hospitals (1%). This organizational time, which is at the expense of clinical time, is correlated with anaesthetists' satisfaction. This result suggests that in public hospitals, and more particularly in university hospitals, there is a difficulty in anticipating the organizational constraints of the operating room and that these latter regulate the work flow in real time. This organizational aspect, through a shift in tasks, is largely the responsibility of the anesthetist who may not be trained to assume this responsibility. Finally, organizational constraints could be a source of conflict between the many different medical professionals working alongside each other in the operating room (surgeons, surgical nurses, nurse anesthetists, nurse assistants).

The time spent on computer media was relatively low (7% of total observation time) compared to other specialties studied in the literature. Indeed, several studies showed that nearly a half of physicians' time was spent on computer tasks (1, 2). In our work, computer media were mainly used in private hospitals compared to public hospitals. In the private hospitals studied, intraoperative processes, including intraoperative monitoring, intra- and post-operative prescriptions and anesthesia consultation, were computerized, while in public hospitals, only post-operative prescriptions were computerized. Unlike other studies, we found a positive correlation between the use of computer media and anaesthetists' job satisfaction (6, 9). This result seems to be in discrepancy with that of another study which showed that the progressive use of electronic devices was rather a source of dissatisfaction for physicians in the U.S (10).

The number of task interruptions in our work was 1.5/h. Savoldelli et al. observed a frequency of distracting events of 5 per induction out of 37 inductions in the context of emergency surgery, the duration of which occupied 35% of the total induction time (11). In another work in the perioperative period, Campbell et al. followed 30 procedures during which they observed 13.8 distracting events per hour (17.4/h during induction, 9/h during maintenance of anesthesia and 30/h during the recovery phase) (12). Finally, out of 32 procedures, Jothiraj *et al*. observed 60 distractor events per hour of which 19.2 scored 2 on the Heavey scale (13). The difference observed between our study and these different works is the definition of task interruption. These other studies evaluated all the events likely to interfere with the physician's vigilance (distraction, disruption and interruption) whereas we only considered those that were responsible for cessation of activity, which reduced the number of our observations. In the context of critical care, several studies have focused on the quantification of interruption with a similar definition to ours. A work by Berg *et al*., published in 2016, found a rate of 5/h in a population of physicians and nurses in an Emergency Department (14). A recent work by Li et al. evaluated task distribution and associated interruptions in an intensive care unit. The time spent in contact with the patient was only 16%, and 4.2/h of task interruptions were recorded In comparison, in a population of specialists not involved in critical care, Westbrook et al. found a 15% rate of patient contact time and a number of task interruptions of 2.9/h (8, 15). These task interruptions had consequences on the satisfaction of anesthetists since we found a correlation between their dissatisfaction and the number of task interruptions per day. Similarly, the work of Berg et al. found this result between dissatisfaction and task interruption (14).

Borrowed from aviation, some authors have put forward the concept of a sterile cockpit, particularly for the induction and recovery phases, to limit task interruptions by staying focused on clinical tasks (16). The International Civil Aviation Organization defines this concept as “the entire period during which the crew should not be disturbed except for matters essential to the safety of the aircraft” and in fact implies the restriction of crew members' activities to those that are operationally essential during particularly complex flight phases (take-off, landing,….). Apart from the exclusion of all non-management discussions, this concept also introduces the use of checklists and also a standardization of communication between the different professionals. This concept has not been specifically studied in anesthesia but other disciplines have shown interest in it. In a before/after study in cardiac surgery, the authors showed that formalizing the elements of communication made it possible to reduce the number of task interruptions (7.3 compared to 11.5 per case) (17). Without necessarily seeking total silence in the operating theater, simple measures such as banning unnecessary movement of people, reducing background noise, temporarily diverting telephone calls can create a more serene, professional and safe atmosphere. The physician (surgeon or anaesthesist) can also ask team members to refocus by warning them that a risky phase of a procedure is about to begin (18). Finally, training physicians to perform or manage multiple tasks simultaneously appears to be an interesting way (19, 20).

Our study presents a number of limitations that need to be included in discussion. We conducted a briefing session with external observers before their observation days to standardize the evaluation, but inter-observer variability between the 13 different external observers cannot be excluded. In addition, the choice of anesthetist to be followed during the day was defined by the study investigator a few days earlier without drawing lots from a population of volunteer physicians to be followed. Since the evaluation took place over 7 consecutive hours and because of the multitude of tasks observed, the observers' attention may have fluctuated, the evaluation and the number of task interruptions were probably underestimated compared to reality especially since as described by Hauschild et al., we didn't perform a multitasking analysis (5). Although the anesthetists volunteered to participate in the study, there is a potential bias that there might not be happy with the working environment.

## Conclusion

In this study, most time in the operating room was spent on clinical tasks with a significant contrast between public and private hospitals for organizational time. The enlargement of the scope of the anesthetists could in part lead to anaesthetists' dissatisfaction.

## Data Availability Statement

The original contributions presented in the study are included in the article/supplementary material, further inquiries can be directed to the corresponding author.

## Author Contributions

VC helped in the study conception and design, in resident recruitment, in study coordination, in interpretation of data, and in manuscript draft and revision. EB helped in the study conception and design, in acquisition of data, in analysis and interpretation of data and in manuscript draft. TC, NB, FL, FJ, NS, JH, and BD helped in interpretation of data and manuscript revision. TE helped in the study conception and design, in acquisition of data, in statistical analysis, in analysis and interpretation of data and in manuscript draft. All authors read and approved the final manuscript.

## Funding

Support was provided solely from institutional and/or departmental sources.

## Conflict of Interest

The authors declare that the research was conducted in the absence of any commercial or financial relationships that could be construed as a potential conflict of interest.

## Publisher's Note

All claims expressed in this article are solely those of the authors and do not necessarily represent those of their affiliated organizations, or those of the publisher, the editors and the reviewers. Any product that may be evaluated in this article, or claim that may be made by its manufacturer, is not guaranteed or endorsed by the publisher.
